# Deciphering the Human Virome with Single-Virus Genomics and Metagenomics

**DOI:** 10.3390/v10030113

**Published:** 2018-03-06

**Authors:** Maria José de la Cruz Peña, Francisco Martinez-Hernandez, Inmaculada Garcia-Heredia, Mónica Lluesma Gomez, Òscar Fornas, Manuel Martinez-Garcia

**Affiliations:** 1 Department of Physiology, Genetics, and Microbiology, University of Alicante, 03690 Alicante, Spain; mj.delacruz@ua.es (M.J.d.l.C.P.); franmhlab@gmail.com (F.M.-H.); garciah.inma@gmail.com (I.G.-H.); m.lluesma@ua.es (M.L.G.); 2 Flow Cytometry Unit: Pompeu Fabra University (UPF) and Centre for Genomic Regulation (CRG), The Barcelona Institute for Sciences and Technology (BIST), 08003 Barcelona, Spain; oscar.fornas@upf.edu

**Keywords:** single-virus genomics, viral metagenomics, oral cavity, bacteriophage, virus, saliva, human, Streptococcus

## Abstract

Single-cell genomics has unveiled the metabolic potential of dominant microbes inhabiting different environments, including the human body. The lack of genomic information for predominant microbes of the human body, such as bacteriophages, hinders our ability to answer fundamental questions about our viral communities. Here, we applied single-virus genomics (SVGs) to natural human salivary samples in combination with viral metagenomics to gain some insights into the viral community structure of the oral cavity. Saliva samples were processed for viral metagenomics (*n* = 15) and SVGs (*n* = 3). A total of 1328 uncultured single viruses were sorted by fluorescence-activated virus sorting followed by whole genome amplification. Sequencing of 24 viral single amplified genomes (vSAGs) showed that half of the vSAGs contained viral hallmark genes. Among those bona fide viruses, the uncultured single virus 92-C13 putatively infecting oral Streptococcus-like species was within the top ≈10 most abundant viruses in the oral virome. Viral gene network and viral metagenomics analyses of 439 oral viruses from cultures, metagenomics, and SVGs revealed that salivary viruses were tentatively structured into ≈200 major viral clusters, corresponding to approximately genus-level groupings. Data showed that none of the publicly available viral isolates, excepting an Actinomyces phage, were significantly abundant in the oral viromes. In addition, none of the obtained viral contigs and vSAGs from this study were present in all viromes. Overall, the data demonstrates that most viral isolates are not naturally abundant in saliva, and furthermore, the predominant viruses in the oral cavity are yet uncharacterized. Results suggest a variable, complex, and interpersonal viral profile. Finally, we demonstrated the power of SVGs in combination with viral metagenomics to unveil the genetic information of the uncultured viruses of the human virome.

## 1. Introduction

Eukaryotic viruses have a direct impact on human health [1], while prokaryotic viruses are thought to influence human body homeostasis by affecting bacterial community structure and function [2,3,4,5]. However, despite viruses being a major component of the human microbiome [4], they have been largely understudied [6,7,8,9]. The discovery of new viruses, such as bacteriophages, has been traditionally approached through the establishment of cultivable virus–host systems. However, culturing methods inefficiently capture naturally occurring viral genomic diversity, especially in prokaryotic viruses, and nowadays viral genomics is moving into the era of metagenomics [10,11], which overcomes some of the culture limitations. A major challenge of viral metagenomics (hereinafter referred to as viromics) is the delivery of accurate and complete viral genomes from viral assemblages. In general, viromics struggles to assemble genomes from viral populations with <2–5× coverage, high microdiversity, and/or uneven abundance [12,13]. A complementary method to viromics is the recent single-virus genomics (SVGs) [14] that disentangles the genetic complexity of viral communities by sequencing the genome of one virus at a time, directly sorted out from natural samples [12,15,16]. Recently, by employing SVGs, we were able to unveil abundant and cosmopolitan marine viruses that have been overlooked by metagenomics and culturing [12].

In the human microbiome, single-cell genomics (SCGs) has indeed been key in sequencing the genome for some of the “most wanted prokaryote taxa” [17]. In microbial ecology, the lack of genomic information for predominant microbes hinders our ability to answer fundamental biological questions about microbial communities. Thus, the implementation of complementary techniques to culture and metagenomics that aid in unveiling the extant genomic natural viral (micro)diversity is paramount in closing that gap. Over the last few years, metagenomics has characterized the viral diversity at different body sites [11,18]. The oral cavity represents an excellent model to apply SVGs, since it is one of the most densely-populated habitats of the human body, with ≈10^8^ virus-like particles (VLPs) per mL of saliva [18]. In this study, we demonstrate the feasibility of using SVGs to sequence the genome of uncultured DNA viruses present in human saliva, and we combine the power of viral metagenomics and SVGs to study the human salivary virome.

## 2. Methods

### 2.1. Human Sample Collection and Processing for Single-Virus Genomics and Viromics

Saliva samples (≈5 mL) were obtained from 15 volunteers (Table S1) with good overall periodontal health, who had signed an informed consent form indicating her/his willingness to participate in this study. Two different methodologies, viromics and single-virus genomics, were applied in this study to recover genome fragments from uncultured viruses present in saliva. Specifically, saliva samples from two volunteers—samples SV92 and SV97—out of 15 were processed in parallel by both approaches: viromics and single-virus genomics. The rest of the salivary samples were processed by viromics (Table S1). Samples were collected in the morning of January 18, 2016, prior to breakfast and oral hygiene, and immediately brought to the laboratory in ice. Then, samples were vortexed for 2 min at maximum speed, centrifuged at 4800× *g* for 10 min at 4 °C, and finally the supernatant (≈4.5 mL) was collected for viromics (4 mL) and single-virus genomics (0.5 mL).

For single-virus genomics, 500 µL of supernatant was diluted in 2 mL of sterile TE buffer (10 mM Tris, 1 mM Ethylenediaminetetraacetic acid (EDTA); pH 8.0) previously filtered through a 0.02 µm Anotop filter (ref. 6809-1002, Whatman, Maidstone, UK). Then, diluted saliva was vortexed for 30 s and filtered through a 0.45 um syringe PES membrane filter (ref. SLHP033RS, Millipore, Milford, MA, USA), vortexed again for 30 s, and finally filtered through a 0.22 µm PES syringe filter (ref. SLGP033RS, Millipore, Milford, MA, USA). In addition, the viral sample SV108 was treated with 10 U/mL of Turbo DNase (Thermo Fischer Scientific, Waltham, MA, USA) at 37 °C for 1 h to remove potential free DNA present in saliva. DNase was inactivated according to the manufacturer’s recommendations. Viral staining and fluorescence-activated viral sorting (FAVS) was performed as previously described [12]. In brief, viral samples previously filtered through 0.22 µm membrane filters were concentrated to 50 μL with Nanosep 10 kDa (OMEGA, Pall Life Sciences, Fribourg, Switzerland), and washed with 500 μL of sterile 0.02 µm-filtered TE buffer (10 mM Tris, 1 mM EDTA; pH 8.0) to remove free DNA. The viruses in sterile TE buffer were then stained with SYBR Gold (final concentration of 4×) at room temperature for 20 min in the dark, and washed three times with 500 μL of sterile 0.02 µm-filtered TE buffer in ultracentrifugal devices. Finally, 500 μL of sterile 0.02 µm-filtered TE buffer was added to the column and recovered for flow cytometry sorting. The whole staining procedure was applied to blanks for flow cytometry analyses as per the recommendation of reference viral staining protocols [19], to identify the correct viral gates for analyses and sorting. FAVS was performed in a BD Influx sorter (Becton Dickinson, San Jose, CA, USA). Reagents and disposable material for sterile FAVS were DNA-decontaminated as described in detail previously [12]. Instrument setup and fine calibration was performed using standard 8-peaks Rainbow beads (Sphero^TM^ Rainbow Calibration Particles 3.0–3.4 μm, ref. 559123, BD Biosciences, Franklin Lakes, NJ, USA,) for laser alignment, and 220 nm 1-peak yellow beads (Sphero^TM^ Nano Fluorescent Particles, Yellow 0.22 μm, Spherotech Inc., Lake Forest, IL, USA, ref. NFPPS-0252-5). “Single” sort mode, which is the most rigorous setting for sorting single particles, was selected for sorting viral particles. The threshold on green fluorescence was set at 1.0 for detecting SYBR Gold fluorescence through a light line passing a 505 LP filter, and collected by a 530/40 nm band-pass filter. All parameters (forward scatter (FSC), side scatter (SSC), and green fluorescence) were collected in logarithmic mode and analysed with BD FACS^TM^ software, version 1.0.0.0.650 (Becton Dickinson, San Jose, CA, USA). Sorting of viral particles was carried out on 384-well plates. Finally, plates were covered with sterile film and stored at −80 °C until used.

The lysis and whole-genome amplification of sorted single viruses from the saliva samples were conducted by applying the conditions as previously described [12]. Essentially, KOH shock in combination with liquid nitrogen shock was used to break the capsid and release the viral DNA, as previously described [12]. Then, whole-genome amplification of the “lysed” sorted viral particles was achieved using multiple displacement amplification (MDA) [12].

For viromics, 4 mL of supernatant was sequentially filtered through 0.45 µm and 0.2 µm filters as above. Free DNA was removed as explained above, using 50 U of Turbo DNase I (Ambion, Invitrogen, Waltham, MA, USA). Finally, viral nucleic acids were extracted with QIAmp^®^ UltraSens^®^ Virus Kit (cat. no. 53704, QIAGEN, Hilden, Germany) according to the manufacturer’s protocol.

### 2.2. Sequencing, Assembly, Annotation, and Genome Analyses

Single-amplified viral genomes (vSAGs) and viromes were sequenced by Illumina technology (Table S1) using the Nextera XT DNA library (ref. FC-131-1024, Illumina*,* San Diego, CA, USA) in a MiSeq sequencer (2 × 250, pair-end), according to the manufacturer’s protocol. Raw reads (Table S1) were quality filtered using the program prinseq-lite [20] with the following parameters: min_length: 50; trim_qual_right: 20; trim_qual_type: mean; and trim_qual_window: 20. Genome assembly was performed with SPAdes version 3.6.1 [21] by applying the following parameters: --sc, -k 33,55,77,99,127, --careful for vSAGs; and metaspades option with parameters -k 33,55,77,99,127 for saliva viromes. Generated vSAGs contigs were subjected to another round of assembly using the Geneious R8 bioinformatic program [22] with stringent conditions (>100% sequence identity and a minimum of 200 bp of overlapping). Then, a thorough inspection was done for all contigs to ensure a nonchimeric assembly. Contigs <2000 bp and contigs matching to human DNA or common bacterial contaminants in single-cell genomics and sequencing [23,24] were removed from the analyses. Contamination screening was done by using a combination of the ProDeGe program and a comparison with the database nr/nt using standalone BLAST version 2.2.31+. Short contigs from a previous salivary survey [18] (maximum length = 349 pb) were reassembled using SPAdes assembler version 3.6.1, with --iontorrent and only-assembler options. General automated annotation was done in metavir [25] and JGI-IMG [26]. Accurate annotation of vSAG 92-C3 was achieved by comparing predicted proteins with the nr database (National Center for Biotechnology Information (NCBI)) using BLAST (Basic Local Alignment Search Tool) version 2.5.0+ [27], and with the pfam database using HMMER (Biosequence analysis using profile Hidden Markov Model) package [28]. Representations of annotated genomes were made using Artemis V12 [29]. Tetranucleotide frequency was calculated with compseq from EMBOSS (European Molecular Biology Open Software Suite) [30]. Principal Component Analysis (PCA) was conducted using FactoMineR package [31] with version R3.2.2. Information for estimating the enrichment of transposase-like genes and other genes was obtained from viromes and microbiomes annotated in the JGI-IMG database [26].

### 2.3. Virome Fragment Recruitment

Virome fragment recruitment was performed to estimate the abundance and ubiquity of saliva vSAGs and other publicly available salivary viruses in databases. We used a database with a total of 439 saliva viral genomes (Table S4 composed of: 6 vSAGs (92-C13, 92-C19, 92-D8, 92-D16, 108-2-M3 and 108-3-D21); 189 viruses inhabiting the oral cavity, obtained from Genbank; 234 contigs obtained by the reassembly of previously published viral contigs [18]; and the ten most abundant contigs assembled from the virome sample SV92. As the query, we used the saliva viromes obtained from the volunteers of this study (Table S1). Fragment recruitment analyses were carried out with standalone Blast version 2.2.31+. The commands used were as follows: blastn -db “viral_database”.fasta -outfmt “6 qseqid sseqid pident length mismatch gapopen qstart qend sstart send evalue bitscore qlen slen” -out recruit_name.blast -query viromes.fasta -evalue 0.00001. Only reads that hit with ≥70% identity and query (read) coverage were considered, and the best hit for each query–subject pair was selected using a modification of BlastTab.best_hit_sorted of Enveomics Toolbox [32]. All hits of every subject were added using R package [31], and the results, in kilobases (Kb), were normalized by dividing by the sequence length (kb) of the viral genome and the virome size (Gb), so that the final result was given as kilobases recruited per kilobase of genome and gigabase of viromes (KPKG), as previously described [12]. Finally, only the viral genomes with ≥40% of coverage were considered.

### 2.4. Gene Content-Based Network Analysis

A total of 424 genome sequences of saliva viruses (Table S4), either complete or partial genomes, were used to build the viral network [33] comprised of the vSAG 92-C13, 189 viruses inhabiting the oral cavity from Genbank, and 234 contigs obtained by the reassembly of viral contigs from a previous salivary virome survey [18]. Proteins were predicted using MetageneAnnotator [34]. All proteins (25,561 proteins) were compared using all-versus-all Blastp (e-value < 1 × 10^−5^), then protein clusters were defined using the Markov clustering algorithm (MCL) [35] with default parameters. Finally, vContact [36] was used to calculate a similarity score between every pair of sequences, and the network was created using Cytoscape (v.3.5.1) [37].

## 3. Results

### 3.1. Single-Virus Genomics: A New Approach in the Context of the Human Virome

**Figure 1:**
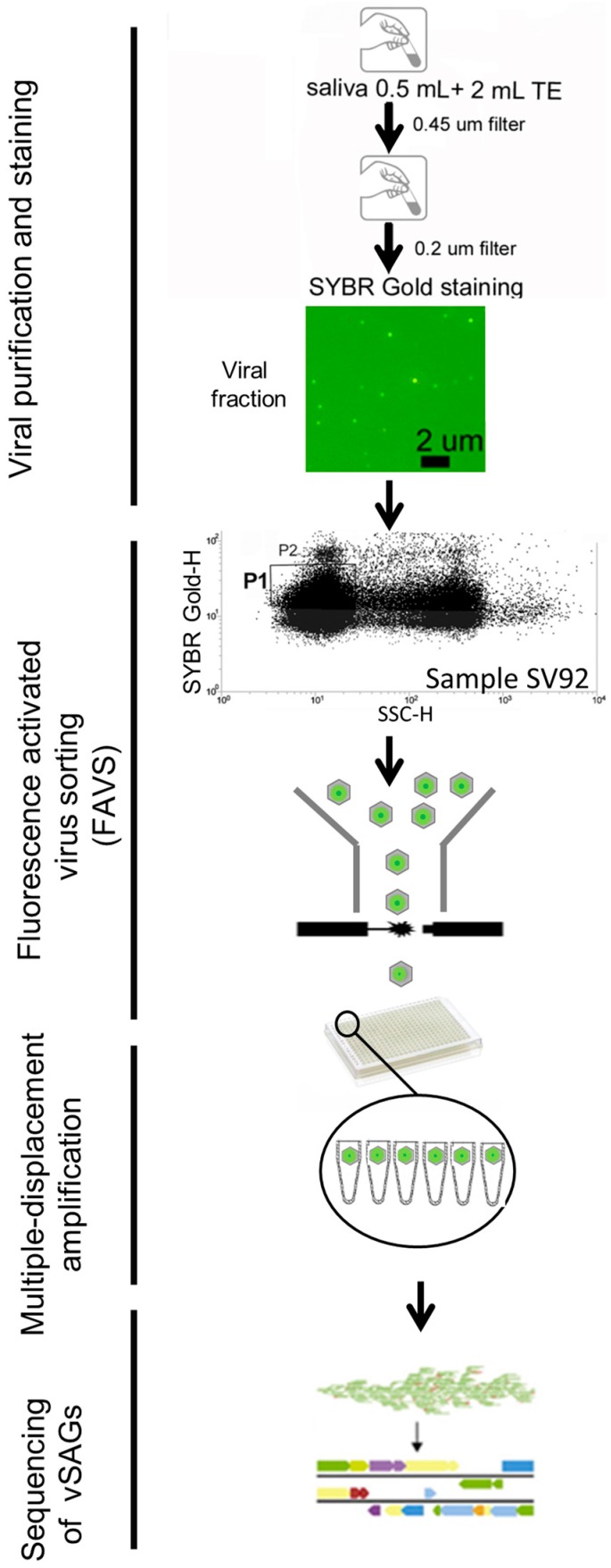
Schematic workflow of single-virus genomics of saliva. Salivary samples were previously filtered to remove eukaryotic cells and bacteria. Viral particles were stained with SYBR Gold. Epifluorescence microscopy imaging of the viral fraction (sample SV92) confirmed the presence of viruses. Single viruses were sorted out by fluorescence-activated virus sorting (FAVS). Flow cytometry plot for the human salivary sample SV92 (see blank in Figure S1): for each sample, the flow cytometric plot of 90° light scatter (SSC-H; height value) and green fluorescence (SYBR Gold-H; height value, relative units) is shown. Gate P1 was used for sorting of single viruses. Viruses of gate P1 clearly outnumbered those in gate P2 (see Figure S1 for more details), and as this study was focused on viruses which are dominant and abundant in the oral cavity, single-virus genomics was performed only on sorted viruses from gate P1. Whole genome amplification of sorted single viruses was achieved by multiple displacement amplification. Positive viral single-amplified genomes (vSAGs) were sequenced by Illumina technology, and further assembled and analyzed.

In our study, saliva was collected from three volunteers (named SV92, SV97 and SV108), and processed in parallel for viromics and SVGs (Table S1). In brief, for SVGs, 0.5 mL of saliva was diluted in 2 mL of TE buffer (10 mM Tris, 1 mM EDTA; pH 8.0), and sequentially filtered through 0.45 μm and 0.22 μm filters to remove cells [38]. Nucleic acids of VLPs were fluorescently stained with SYBR Gold [19], and epifluorescence microscopy confirmed the presence of VLPs (Figure 1). Flow cytometry data showed a clear and distinguished gate of stained virus-like particles discriminated from the noise (Figure 1 and Figure S1), as previously described [19]. Gate P1, as indicated in Figure 1, was used in this study as the sorting gate. In sample SV92, the events obtained in gate P1 (*n* = 28,969, 43% of total events) clearly outnumbered those obtained in blanks (*n* = 623, ≈4% of total detected events; Figure S1). Results from the blanks indicated that events with ≤10^1^ relative units of green fluorescence are likely noise caused by debris or free unbound dye (Figure S1). A total of 1328 VLPs were sorted out in an Influx flow cytometer (BD) sorter [12] (Figure 1 and Table S1). Then, KOH and temperature shock were applied to break down the viral capsids [12,14,39]. Whole genome amplification was performed by multiple displacement amplification (MDA) [12]. Success rate of the MDA ranged between 2 and 22.5% of sorted single viruses (Table S1). A total of 24 vSAGs from the three samples were selected at random for Illumina sequencing (Table S1). As previously described [12], those vSAGs (*n* = 12) that yielded partial and fragmented genome assembly reconstruction (contigs <2 kb) were not considered for further analyses. Genome annotation showed that ≈50% of vSAGs contained viral hallmarks genes (Table S2). The rest of the vSAGs contained an unexpected amount of transposon- and plasmid-like genes without concomitant viral genetic signals (Table S2). Then, we sought to investigate if homolog transposon- and plasmid-like genes were also present in the corresponding viromes. For instance, in the viromes of samples SV97 and SV92, a total of 83 and 65 transposases were detected, respectively, and represented up to ≈1% of the total predicted genes. In line with this, in general, salivary viromes showed one of the highest enrichments of transposase-like genes among a total of 227 analyzed viromes and microbiomes from different environments (Table S3 and Figures S2–S4), ranking fifth within gene categories with known function (≈0.6%, Figures S3 and S4). For some vSAGs, such as 92-D16 and 108-H18, insertion sequence elements (IS5, IS3, IS1, IS1595, and IS1182) common in transposons were also detected (Table S2). Furthermore, some transposases in vSAGs were also found in salivary viromes (Table S3), and were highly related to transposases of the oral bacteria genera *Moraxella* and *Enhydrobacter* (80–90% amino acid identity, Table S3), also detected in the analyzed salivary viromes. Nevertheless, either through single-virus genomics or viromics, data indicated that transposase and other mobile elements are abundant in the 0.2 µm-filtered fraction of saliva.

### 3.2. The Uncultured Abundant Single Virus 92-C13

**Figure 2:**
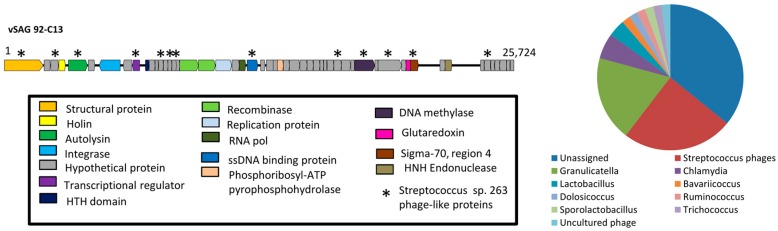
Genome annotation of the sorted single virus vSAG 92-C13. Conserved ORFs within Streptococcus phages are indicated with asterisk (*). The pie chart depicts the closest genera hits of the annotated proteins of vSAG 92-C13 (see Methods for details).

**Figure 3:**
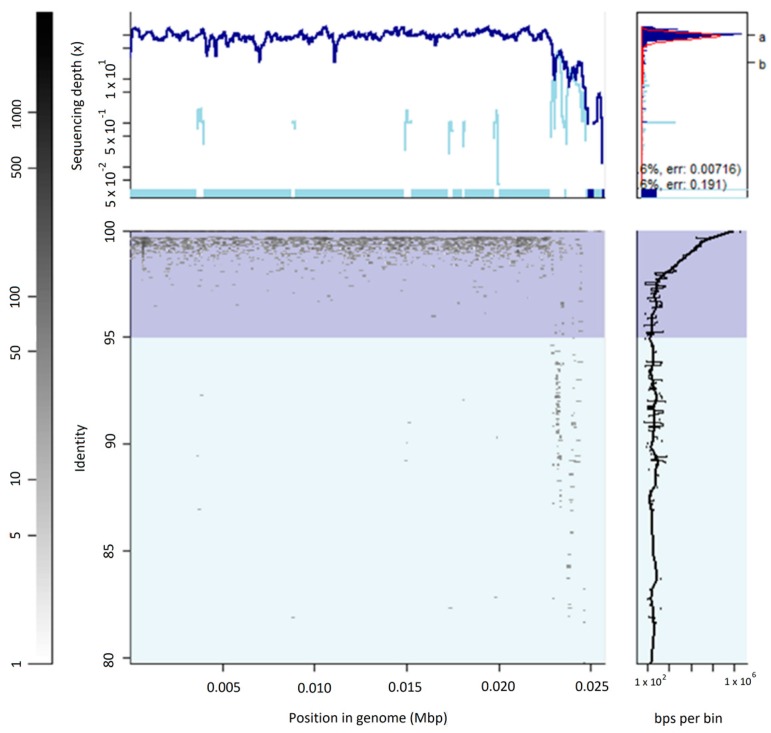
Viral metagenomic fragment recruitment of vSAG 92-1-C13 in the virome sample SV92. This vSAG is fully recruited with reads at 99–100% identity. Sequencing coverage and total recruited nucleotides at different identity percentages are depicted.

**Figure 4:**
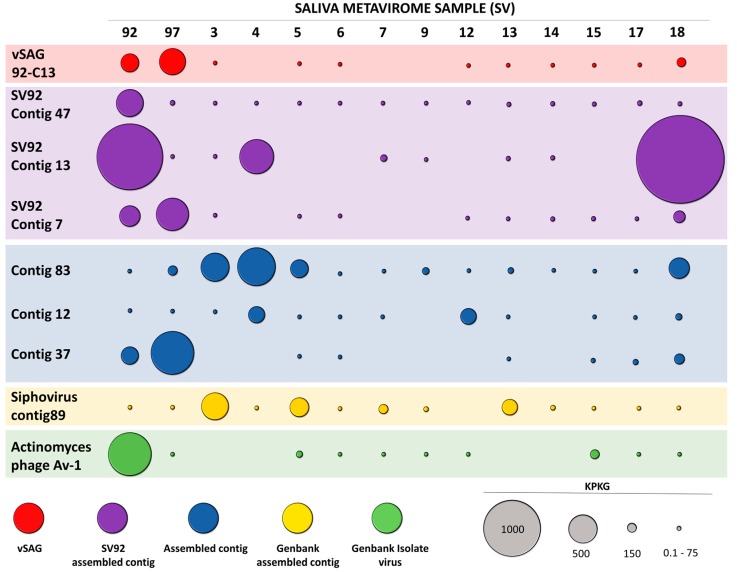
Abundance and ubiquity of viruses from the oral cavity. Virome fragment recruitment of nine of the most abundant oral viruses, obtained using different techniques, is shown. Circle color differentiates the viral genomes obtained from: single-virus genomics (red; this study); assembled data from our salivary virome samples (purple); reassembled data from previously published salivary viromes (blue) [18]; assembled viral contigs by Pride and colleagues [18], and viral isolates from the Genbank database (green). The size of the circle depicts the recruitment rate, expressed in kilobases recruited from each virome (Kb), and normalized by the viral genome length (kb) and the virome size (Gb) (KPKG). Only reads with ≥70% identity and query coverage and viruses with ≥40% coverage (subject coverage) were considered. Absence of a circle indicates no recruitment or recruitment below applied thresholds. As the sequencing reaction for virome sample SV108 failed, there are a total of 14 viromes included in these analyses.

**Figure 5:**
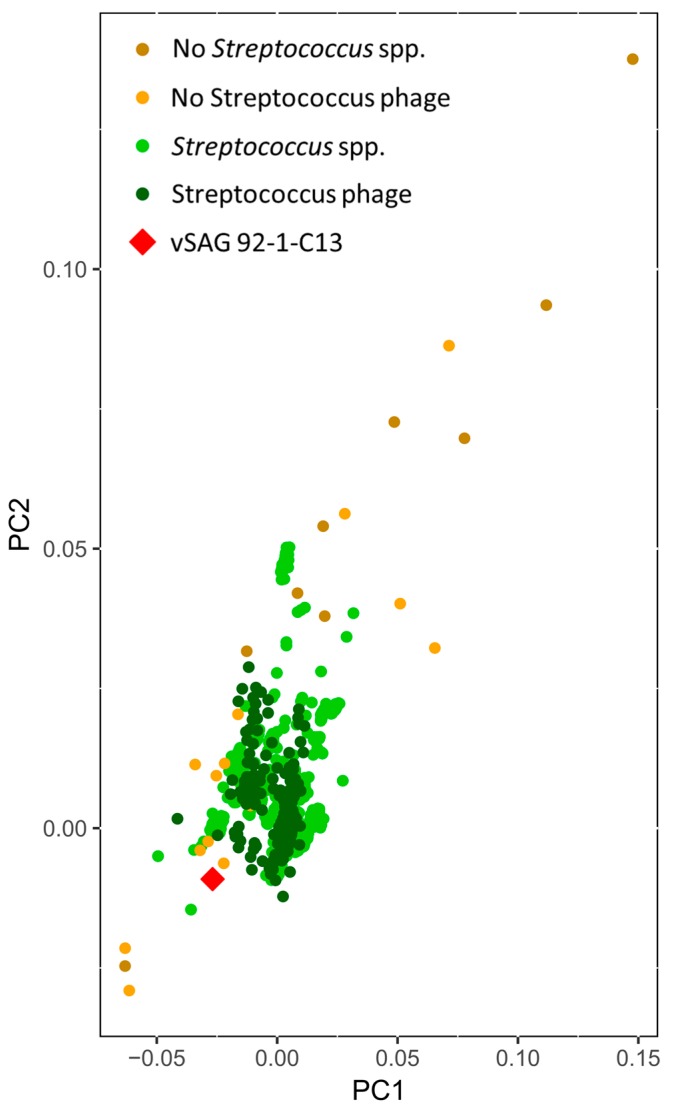
Principal component analysis of tetranucleotide frequencies. For the analysis, we compare 189 oral bacteriophages, 10,751 putative bacterial hosts, and vSAG 92-1-C13, in order to define the connection between vSAG 92-1-C13 and all populations present in the oral cavity.

Among those vSAGs representing bona fide viruses, the vSAG 92-C13 (Figure 2) sorted out from the viral fraction gate P1 of sample SV92 (0.22 μm-filtered, Figure 1) was one of the most abundant viruses in the oral virome (ranking 14 and 2 in samples SV92 and SV97, respectively). This viral genome was fully recruited in the corresponding viral metagenome (Figure 3), and was present in four of the analyzed samples (Figure 4). Nearly 25% of the annotated proteins of that virus were assigned to prophages that infect different *Streptococcus* species (Figure 2), and half of them were assigned to a prophage of *Streptococcus* sp. strain 263 (Figure 2 and Table S5). Although tentative, according to manual genome annotation (Tables S2 and S4) and the Metavir platform, the uncultured virus 92-C13 and the rest of the bona fide viruses (vSAGs 108-D21, 108-M3, 108-G5, 92-C19, 92-D8) could belong to the order *Caudovirales*. Principal component analysis based on genome tetranucleotide frequencies [33] of oral bacteriophages (*n* = 189; Table S4) and putative bacterial hosts (*n* = 10,751, obtained from NCBI and GOLD databases) showed that the abundant virus vSAG 92-C13 was related to a cluster of Streptococcus-infecting phages (Figure 5), albeit not placed at the center of that group.

### 3.3. Abundance and Ubiquity of Viruses in the Oral Virome

Virome metagenomic fragment recruitment was used to assess the abundance and ubiquity of publicly available viruses of the oral cavity in our viral salivary metagenomes. This viral dataset was comprised of 439 oral viruses (Table S4), including 189 publicly available isolates (mainly Streptococcus phages), our single viruses, and 234 assembled viral contigs obtained in this study and by Pride and colleagues [18]. Data showed that none of these 439 oral viruses were present in all analyzed salivary viromes (Figure 4 and Figure S5 and Table S6). Within the top ten most abundant viruses, a clearly uneven abundance was observed among samples, and no apparent predominance of the same viral species was found in the saliva samples (Figure 4 and Figures S5 and S6). For instance, the most abundant and dominant virus in the virome sample SV92 (viral contig 13) was not present in the rest of the samples, except that of the virome 4 (Figure 4). Similar results were found for the abundant vSAG 92-C13, and other salivary viruses. Viral genomes from viromics were clearly more abundant than isolates, and only the Actinomyces phage isolate Av-1 (NC_009643.1), out of 189 isolates, was significantly detected in four of the analyzed viromes, while the rest were almost absent (Figure 4).

### 3.4. Tentative Viral Community Structure of the Oral Cavity

**Figure 6:**
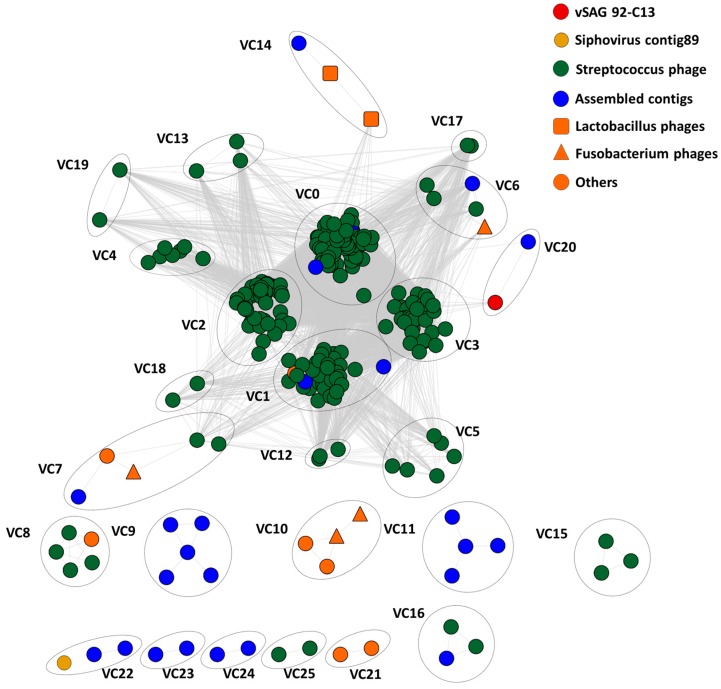
Protein-sharing viral network of oral viruses. (See Supplementary methods for details about the construction of the network.) Approximately genus-level groupings are shown as viral clusters (VCs). The network is built based on the relations (edges) between all proteins of 482 oral cavity viruses. Genomes were from this study, viral isolates were from the Genbank database, and viral contigs were from Pride and colleagues [18] and also from this study. The vSAG 92-C13 (indicated by a red circle) is related to one of the largest Streptococcus phage VCs, the VC3. Note that a total of 210 singlet clusters, each formed by one viral contig, are not depicted in the viral network.

Viral gene network analyses have been recently used to address the relatedness and grouping of viruses within the context of viral taxonomy [12,33,36]. Here, we calculated a massive viral gene network with all publicly available oral viruses (*n* = 424), to unveil the structure of viral communities in the oral cavity (Figure 6). Data revealed that salivary viruses were structured into 26 major homogenous viral clusters, comprised of at least two viruses (see Methods) with a high number of gene-sharing connections. Each viral cluster (VC) corresponds to approximately genus-level groupings, as previously described [12,33,36] (Figure 6). In the case of uncultured viruses from the metagenomic assembly (*n* = 234), a total of 210 viral contigs did not have connections with any other virus, and thus each one of these viral contigs formed a singlet cluster (note that these are not depicted in Figure 6). It is important to remark that the total number of viral clusters in the analyzed samples was ≈200, albeit most of them are comprised of a single viral member. In the case of the characterized Streptococcus phage isolates (*n* = 183) available in the Genbank database, they were grouped in the four largest homogenous viral clusters (VCs) 0–3, or genera with a high number of gene-sharing connections. It is worth noting that among those 26 major clusters with a high number of connections, most of the viral clusters (*n* = 20) have a representative isolate. Finally, in good agreement with the host prediction data of vSAG 92-C13 (Figure 5), this single virus was in a discrete viral cluster (VC20), but was related to VC3, a prominent and the largest Streptococcus phage viral cluster found in the oral virome, along with VC0, VC1, and VC2.

## 4. Discussion

SVGs is a novel tool in viral ecology [12,14,15,16], not exempted from technical challenges, similar to those for SCGs. SVGs is susceptible to technical limitations, which could explain, for instance, the unexpected low MDA success rate obtained for the sample SV92; ≈10-fold reduced compared with the other saliva samples or the marine samples analyzed in our previous study [12]. Thus, data suggest that whole genome amplification steps here were less robust. Recently, a new and more efficient thermostable mutant phi29 polymerase has greatly improved genome coverage and whole genome amplification performance in single-cell genomics [15], and thus its application in SVGs is promising. Our study also highlights that SVGs pipeline optimization is likely necessary for particular environments. Saliva, unlike other type of samples such as seawater [12], is chemically and biologically more complex, with a higher amount of extracellular genetic material, and thus DNase treatment is always recommended. In saliva, we speculate that recalcitrant bacterial DNA could remain in the DNA-treated sample. The origin for those vSAGs obtained here with an unexpected level of transposon- and plasmid-like bacterial genes is uncertain. Here, by employing both approaches of viromics and SVGs, data showed that mobile elements such as transposases are, unexpectedly, very abundant in salivary viromes. These results open the possibility of the sorting of genetic transfer agents (GTAs) [40] or even the extracellular vesicles [41] present in saliva that may be released by oral bacteria. Although a mere bacterial contamination from free DNA, or DNA recalcitrant to DNase treatment cannot be ruled out, it is worth noting that vSAGs with those genetic features were also found in the DNase-treated samples and in the corresponding viral metagenomes obtained in this study.

The detection of viral particles in saliva with very small genomes, such as ssDNA and RNA viruses, remains complicated with current staining protocols and flow cytometers. Advances in micro- and nanofluidics, and new dedicated flow cytometry sorters tuned for targeting small particles, such as vesicles and viruses, are urgently needed. In this study, viruses with a diameter larger than 0.22 μm were not considered. Flow cytometry data indicated the presence of two viral populations, based on side scatter and fluorescence signals (gates P1 and P2, Figure 1). Salivary viruses belonging to the gate P1 population clearly dominated the community, while population gate P2 constituted a minor fraction. The viruses detected in gate P2 with higher fluorescence signals may have larger genomes. In this study, gate P2 was not analyzed by SVGs since it only represented <1% of the total viral community, and here we focused on the abundant members of the virome community.

Nevertheless, by employing the novel SVG approach, we have discovered a total of eight new viruses (Tables S1 and S2), including the vSAGs 92-C13, that are naturally abundant in saliva (Figure 3); a great example of the power of this new methodology. In the particular case of this virus, the fact that it is closely connected to Streptococcus phages (Figure 5 and Figure 6), but separated from the center of cluster cores leads to speculations that it could infect non-Streptococcus bacteria as well.

In our study, we have not addressed the diversity and community structure of human and RNA viruses of the oral cavity, but have focused on bacteriophages putatively infecting prokaryotes. Our results, regarding viral community structure, support the concept of a variable and interpersonal pool of viruses, which is in line with those previously published by Abeles and colleagues [38]. Here, in addition, we employed a new approach based on viral gene content network analysis that intends to unveil the structure of viral populations present in the analyzed samples. This recent approach has been proved very powerful to gain insight into the diversity and structure of a viral community [12,33,36]. We anticipate that our estimation of viral clusters is conservative, and it is likely that more viral clusters will be discovered with higher sequencing coverages. The biological meaning of the singlet viral clusters found needs further investigation, but it is tempting to speculate that these viral ‘populations’ are formed by a single species with no other close viral relatives. Viral taxonomy remains complicated, but recent surveys based on viral networking analyses showed good concordance with current taxonomy [23]. It is striking that according to metagenomic fragment recruitment data, the concentration of viral isolates was very low in the analyzed viromes, and thus they represent subdominant members of the community. Previous viral metagenomic surveys demonstrated that the oral cavity hosts a persistent, robust, and gender-consistent viral community [18,36]. Furthermore, they showed that one of the obtained viral contigs (number 86) was fairly abundant in the analyzed samples. Here, we expanded those analyses by employing multidisciplinary omics approaches, and performing a massive viral metagenomic comparison with all publicly available oral viruses, from cultures and metagenomics, to assess their natural abundances.

## 5. Conclusions

Our results provide evidence of the potential of SVGs for complementing current methods in viromics to unveil the extent of viral genetic (micro)diversity in the human virome, and aid in understanding the viral community structure of the human virome. The employed SVGs methodology can be implemented with any liquid samples from the human body. Here, by employing SVGs, we have been able to unveil the genome of the vSAG 92-C13, and other uncultured viruses that were predominant members in the analyzed salivary viromes. They were more abundant than many of the viral isolates obtained to date. Overall, most of the available viral isolates are likely to be rare members of the community, and do not represent the predominant viruses of the human salivary virome. Furthermore, metagenomic data does not support a simple salivary viral core. In contrast, a more variable, complex, and interpersonal viral profile in saliva was observed. Finally, viruses of oral viromes were structured in ≈200 major clusters, of which 26, represented mostly by Streptococcus phages, showed high number of gene-sharing connections.

## Supplementary Materials

The following are available online at http://www.mdpi.com/1999-4915/10/3/113/s1. Figure S1: Flow cytometry for the human salivary sample SV92; Figure S2: Gene frequency of detected and annotated transposase and transposase-like genes in viromes and microbiomes available at JGI-IMG platform; Figure S3: Gene frequency of typical viral hallmark genes in viromes and microbiomes used in Figure S2; Figure S4: Total counts of most abundant annotated genes with known function from all analyzed salivary viromes obtained in this study; Figure S5: Abundance of oral cavity viruses in the analyzed viromes; Figure S6: Percentage of recruited virome for each one of those abundant viruses depicted in Figure 4; Table S1: Information of salivary samples processed for viral metagenomics and single virus genomics; Table S2: Genome annotation of single-viruses; Table S3: Comparison of transposases found in single-viruses and viral contigs from salivary viromes obtained in this study; Table S4: Viruses used in Virome fragment recruitment (VFR), gene-content based network (GN) and PCA analysis (PCA); Table S5: Summary table of BLAST and PFAM annotation results for the vSAG 92-C13; Table S6: Virome fragment recruitment data.

## Availability of Data and Materials

Genomes of vSAGs from this study are available in the Supplementary material. Viral contigs and annotation of salivary viromes are available at the JGI-IMG platform (IMG Identifiers: 3300009952-3300009965; see Table S1 for details). In addition, all datasets used and/or analysed during the current study are available from the corresponding author on reasonable request.
