# Supplementary material for: Deciphering the Human Virome with Single-Virus Genomics and Metagenomics
Source: Viruses. 2018 Mar 6;10(3):113. doi: 10.3390/v10030113 (PMC5869506; doi:10.3390/v10030113)
Supplement: Supplementary file 1 [file viruses-10-00113-s001.zip › Supplementary Information/Fig S1.docx]

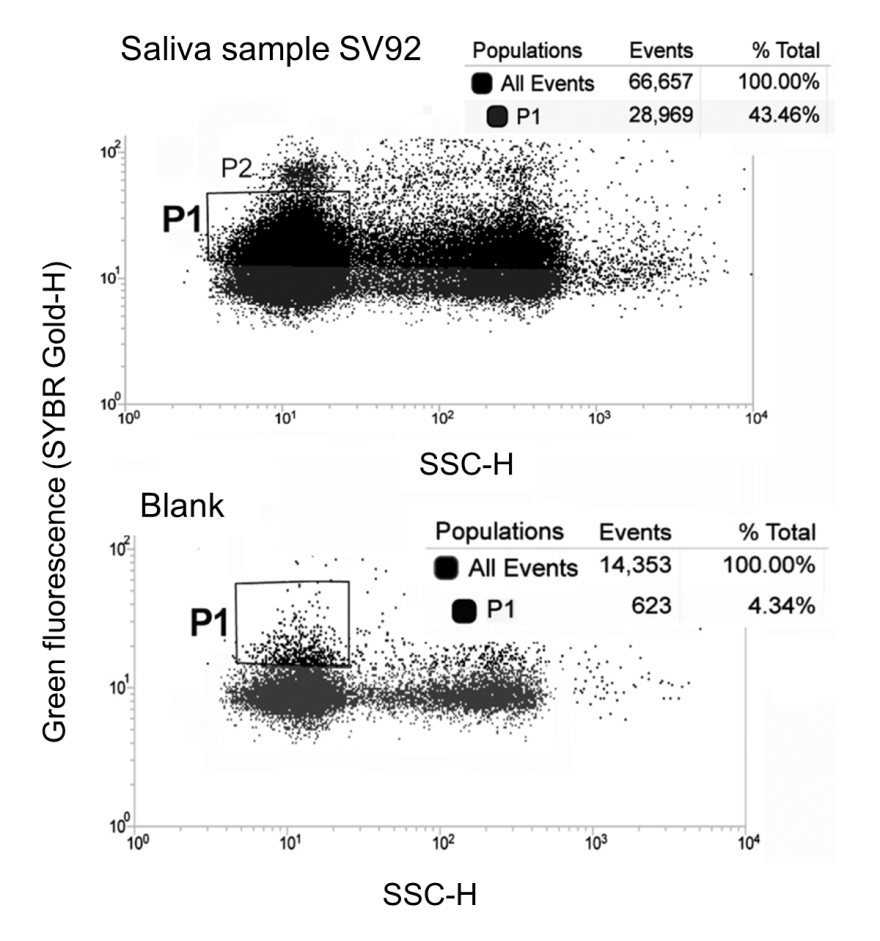


**Fig. S1.** Flow cytometry plot for the human salivary sample SV92 and blank. For each sample, flow cytometric plot of 90° light scatter (SSC-H; height value) and green fluorescence, (SYBR Gold-H; height value, relative units) is shown. Gate P1 was used for sorting of single-viruses. Blanks were processed as previously described [12]. Note that events in gate P1 of blank only represent <2% of total events detected in gate P1 of sample SV92. These events detected in blanks are likely electronic noise as previously discussed [12] or remaining free SYBR Gold dye in the sample after washing steps.
