# Supplementary material for: Deciphering the Human Virome with Single-Virus Genomics and Metagenomics
Source: Viruses. 2018 Mar 6;10(3):113. doi: 10.3390/v10030113 (PMC5869506; doi:10.3390/v10030113)
Supplement: Supplementary file 1 [file viruses-10-00113-s001.zip › Supplementary Information/Fig S2.docx]

**
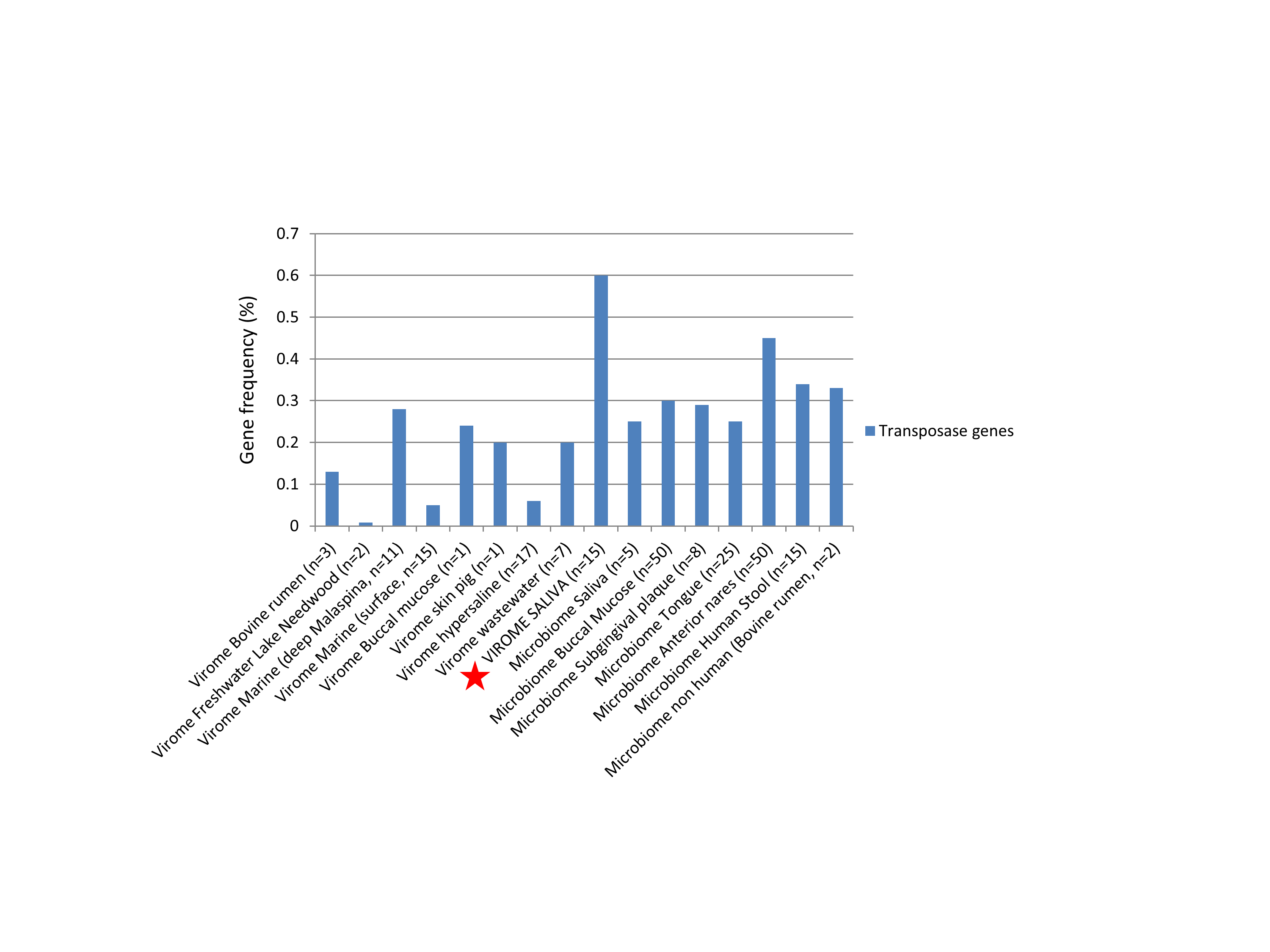
**

**Fig. S2.** Gene frequency of detected and annotated transposase and transposase-like genes in a total of 227 viromes and microbiomes available at JGI-IMG platform. Salivary viromes of this study are indicate with a star. Frequency was obtained by dividing number of genes annotated as transposase and transposase-like genes by the total detected genes. The search at JGI-IMG pipeline platform was used with the word “transposase”.
