# Supplementary material for: Deciphering the Human Virome with Single-Virus Genomics and Metagenomics
Source: Viruses. 2018 Mar 6;10(3):113. doi: 10.3390/v10030113 (PMC5869506; doi:10.3390/v10030113)
Supplement: Supplementary file 1 [file viruses-10-00113-s001.zip › Supplementary Information/Fig S3.docx]

**
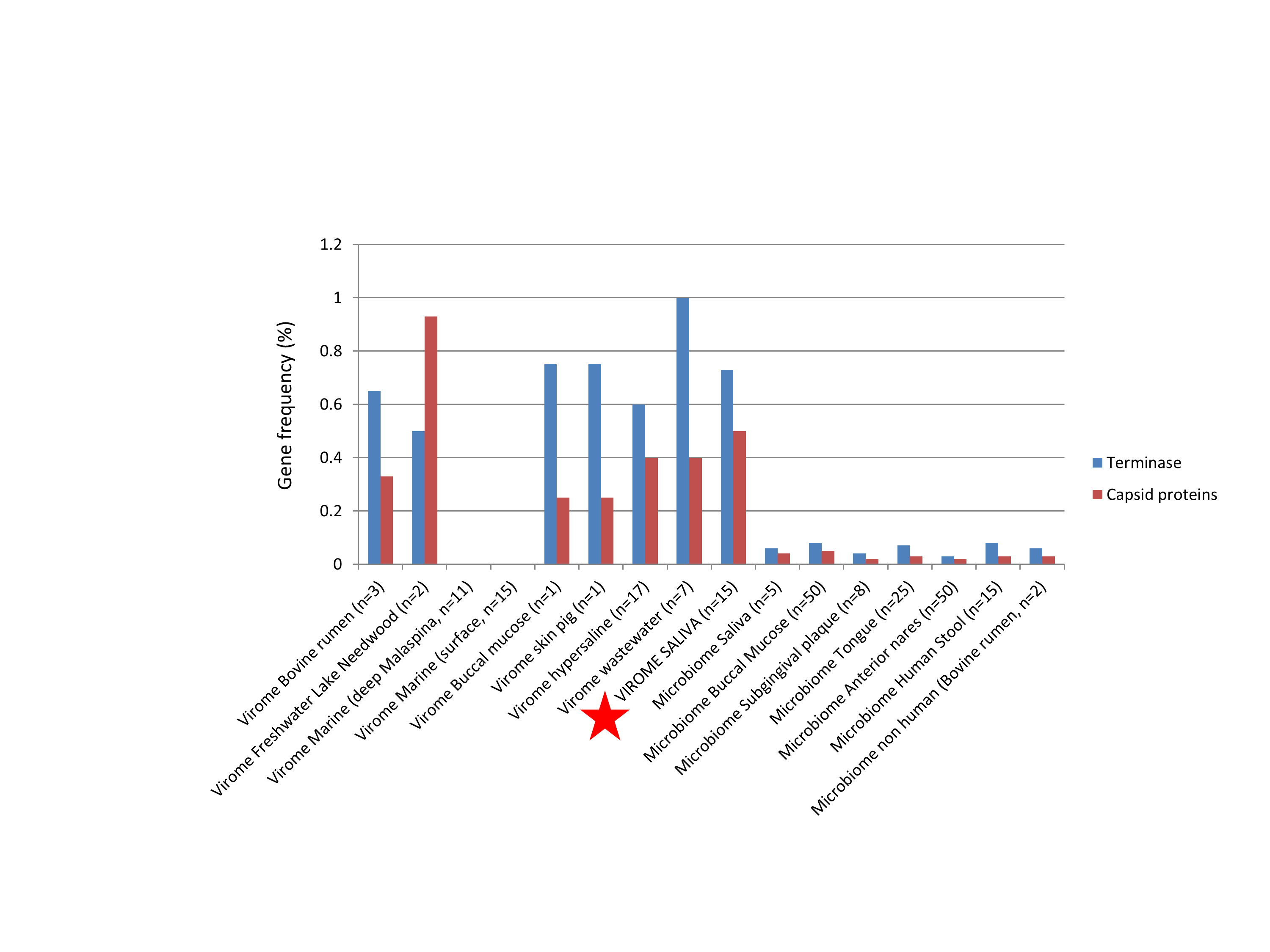
**

**Fig. S3.** Gene frequency of typical viral genes in 227 viromes and microbiomes used in Fig. S1. As expected, viromes contained higher viral gene frequencies of terminases and capsid proteins confirming that they were clearly enriched in viral particles with low levels of bacterial DNA, while microbial metagenomes showed very low frequency. An exception was those viromes from Malaspina expedition that contained similar features as microbial metagenomes due to high contamination with bacterial DNA (personal communication by Simon Roix and Matt Sullivan). The search at JGI-IMG pipeline platform was used with the words “terminase” and “Capsid”.
