# Supplementary material for: Deciphering the Human Virome with Single-Virus Genomics and Metagenomics
Source: Viruses. 2018 Mar 6;10(3):113. doi: 10.3390/v10030113 (PMC5869506; doi:10.3390/v10030113)
Supplement: Supplementary file 1 [file viruses-10-00113-s001.zip › Supplementary Information/Fig S4.docx]

**
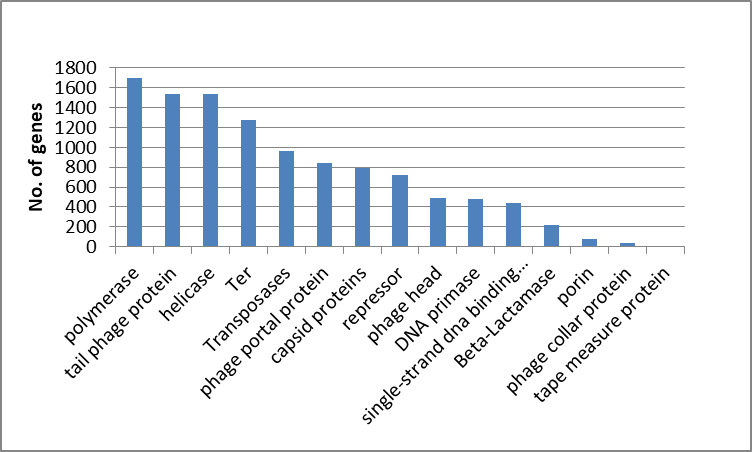
**

**Fig. S4.** Total counts of most abundant annotated genes with known function from all analyzed salivary viromes obtained in this study. Total counts are the sum of genes from all viromes for each gene category. Viral contigs were assembled and annotated at JGI-IMG.
