# Supplementary material for: Deciphering the Human Virome with Single-Virus Genomics and Metagenomics
Source: Viruses. 2018 Mar 6;10(3):113. doi: 10.3390/v10030113 (PMC5869506; doi:10.3390/v10030113)
Supplement: Supplementary file 1 [file viruses-10-00113-s001.zip › Supplementary Information/Fig S5.docx]

SV5

SV4


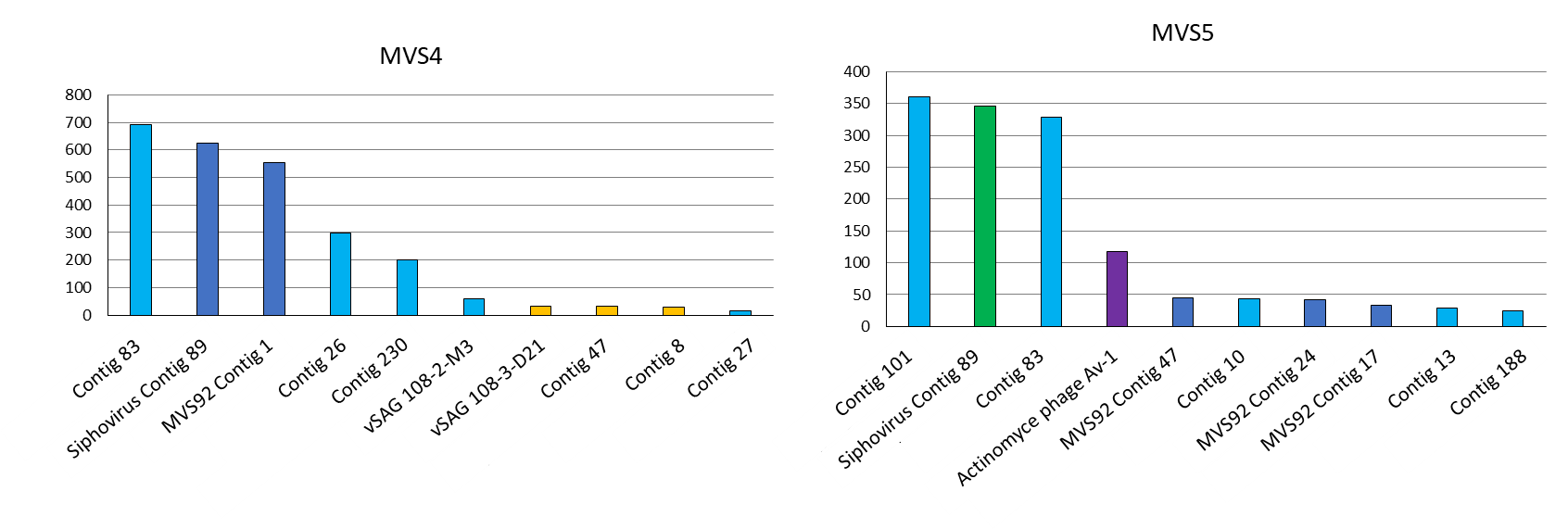


KPKG


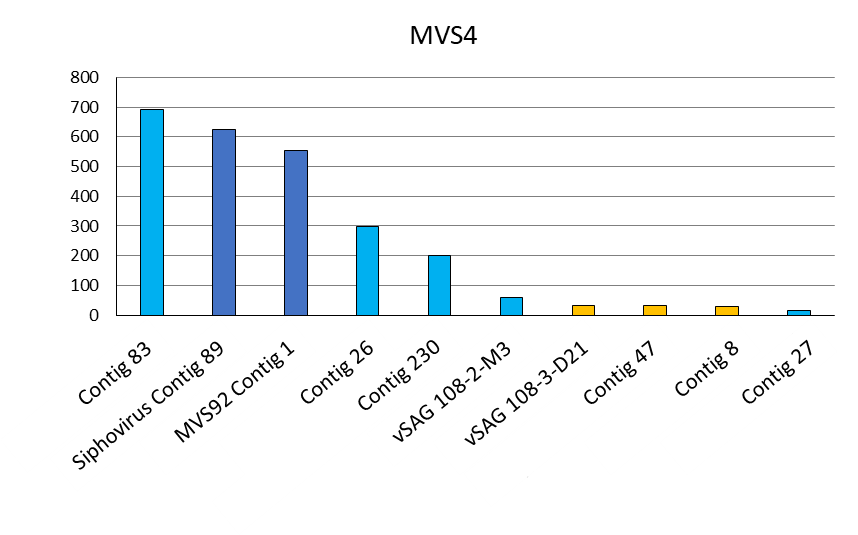


KPKG

SV7

SV6

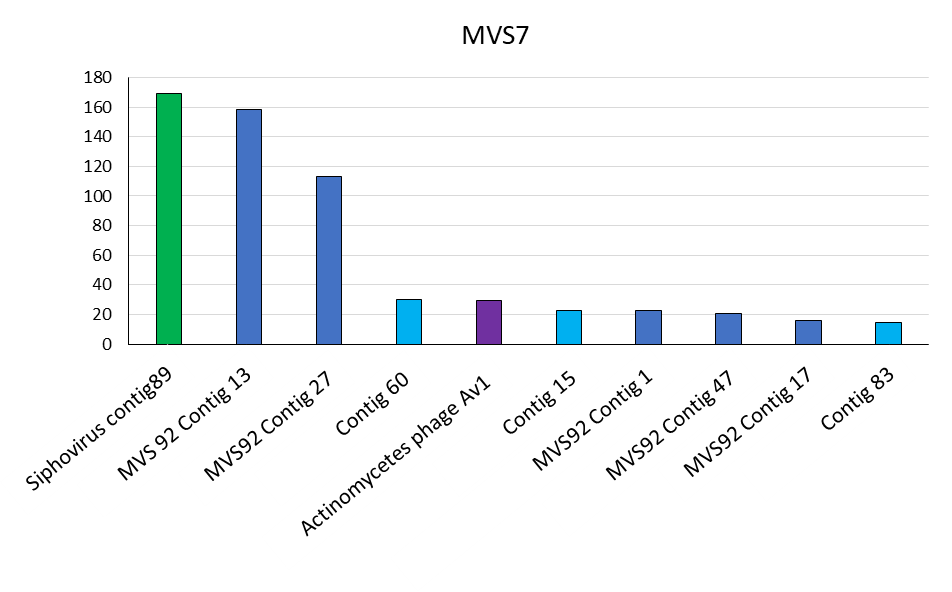


KPKG


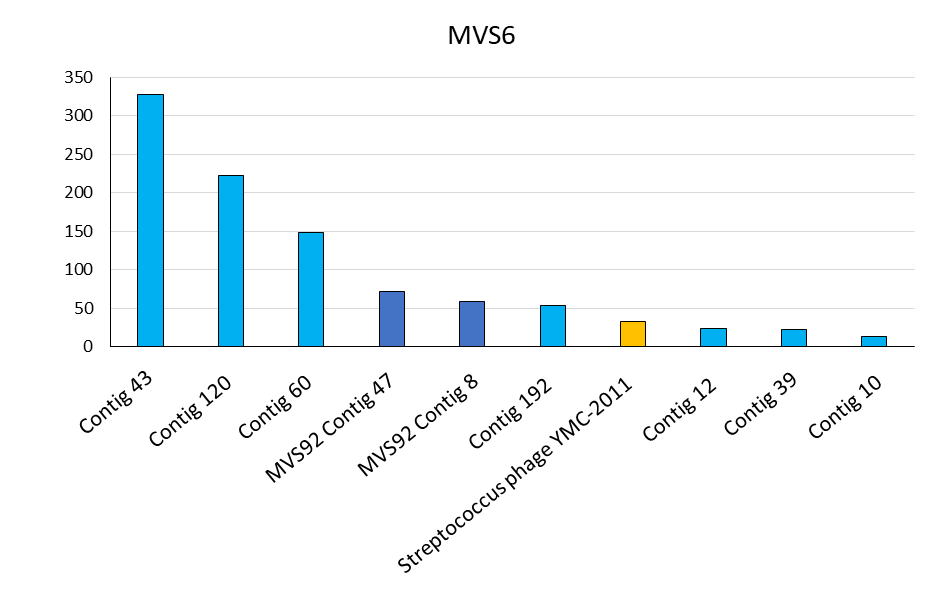


KPKG

SV9

SV97

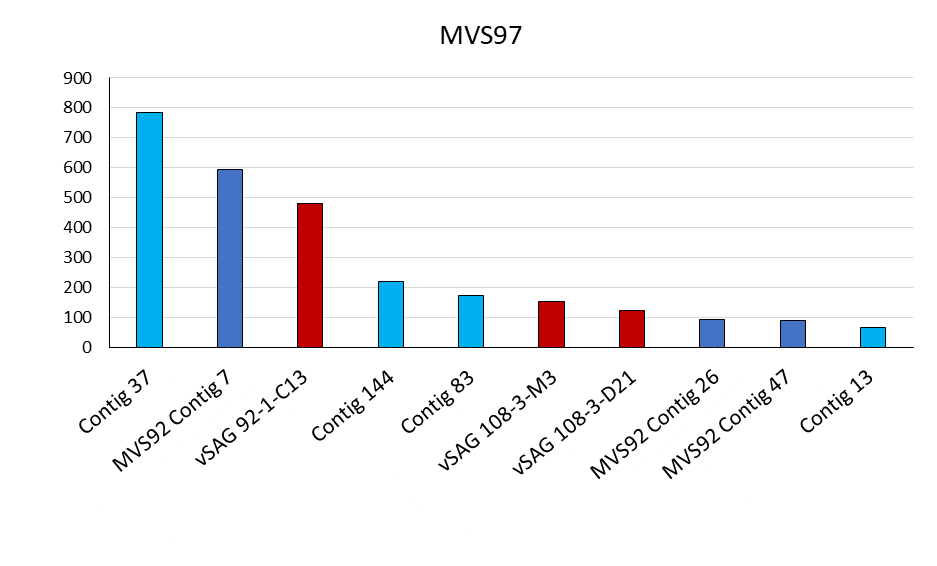


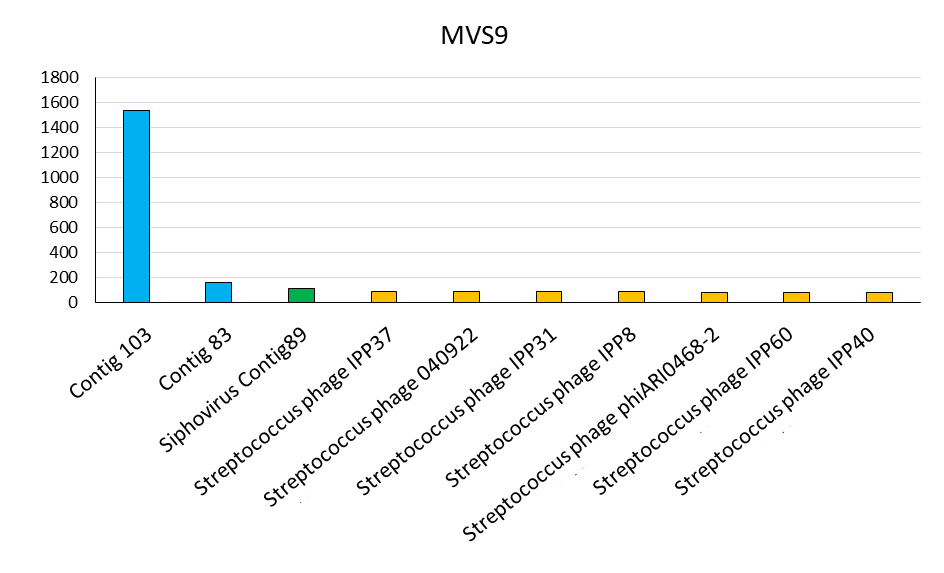


KPKG

KPKG

SV135

SV125

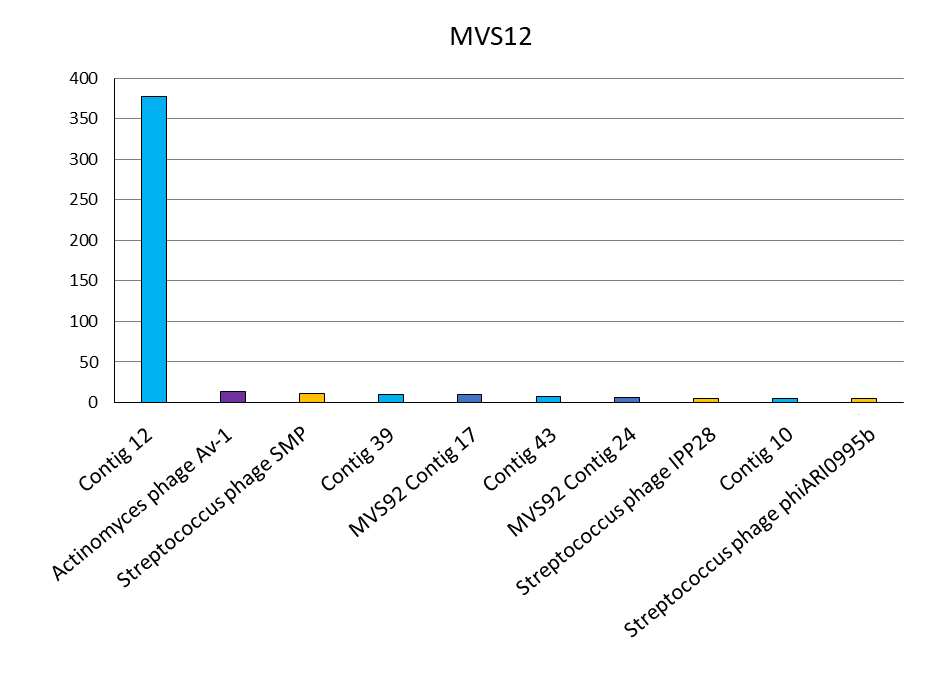


KPKG


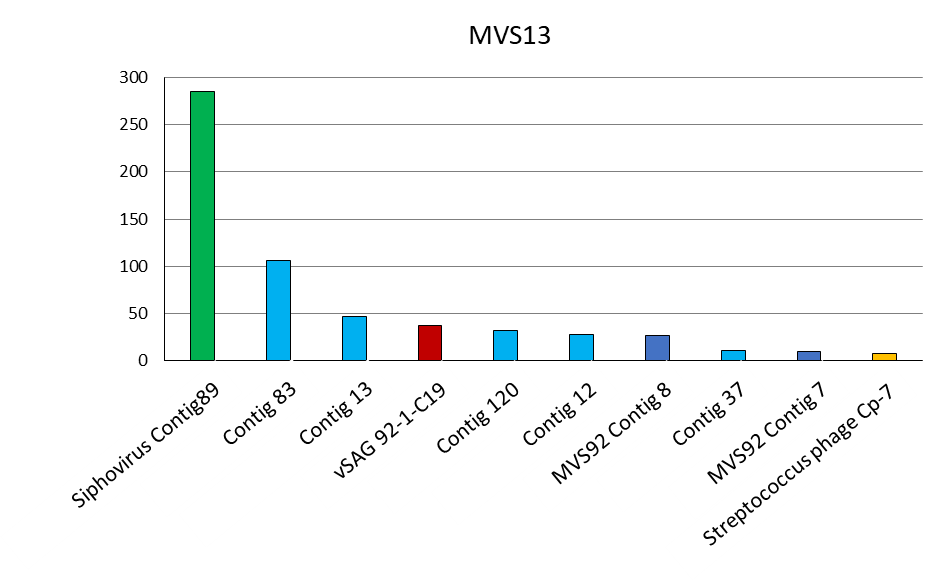


KPKG

SV155

SV145

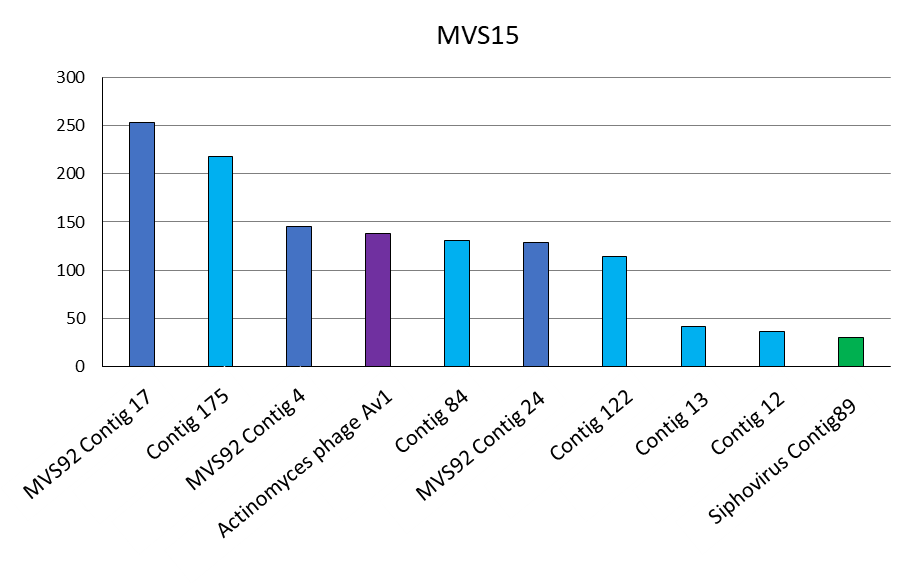


KPKG


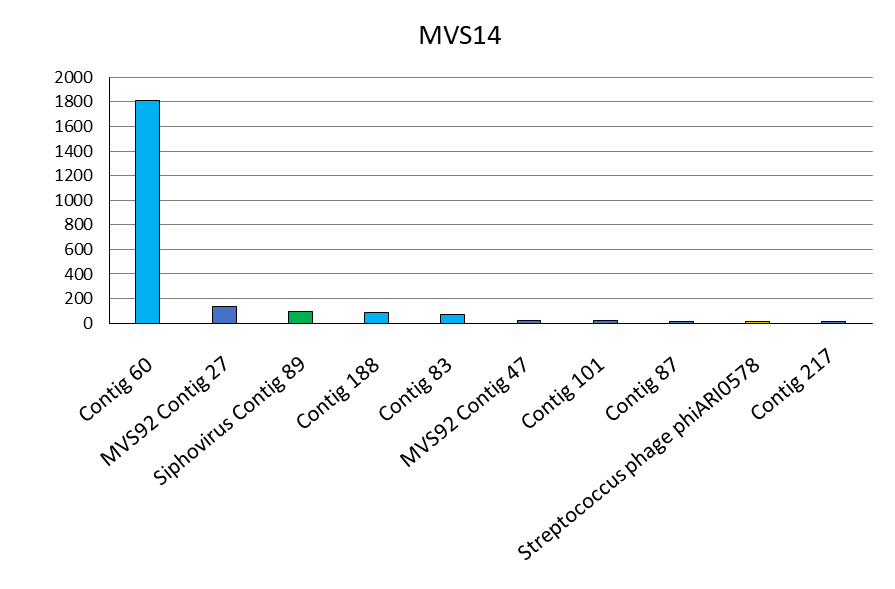


KPKG

SV185

SV175

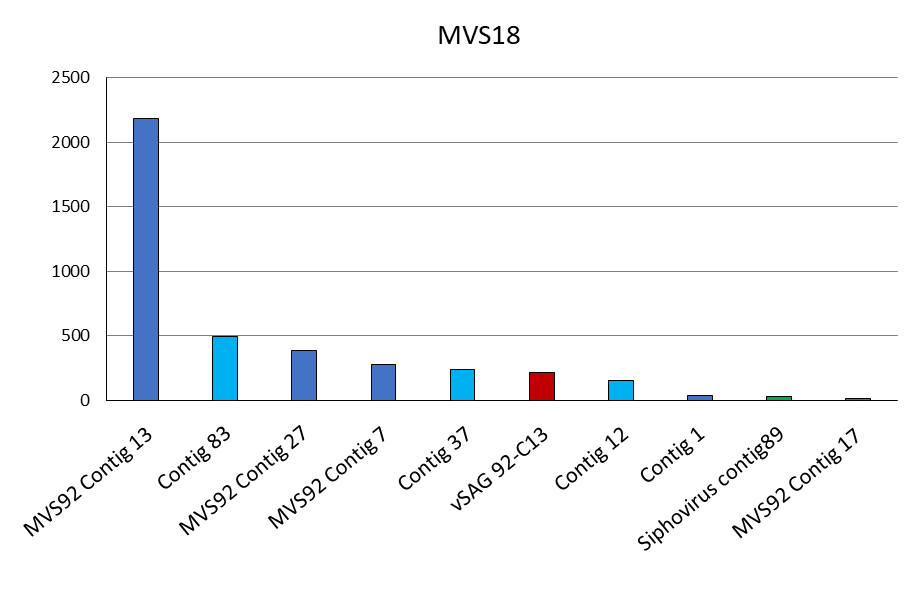

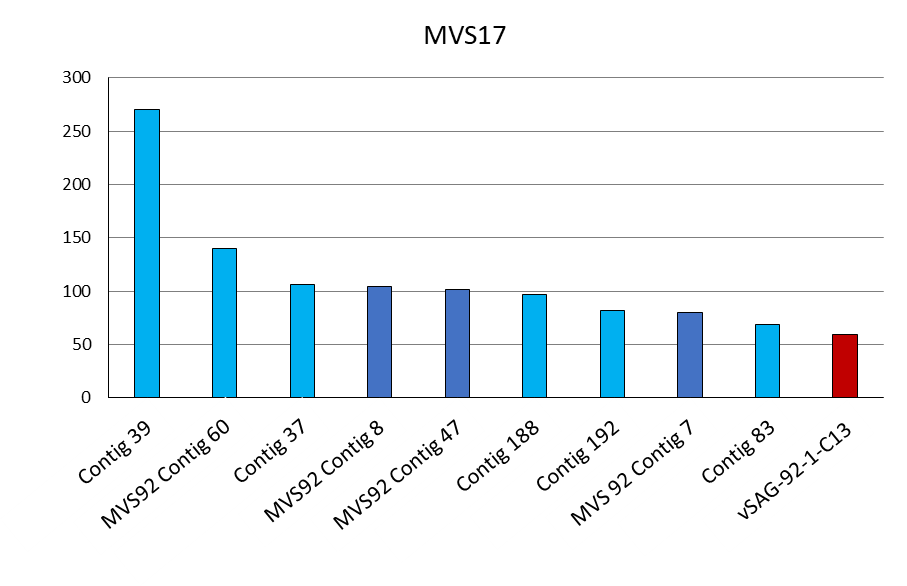


KPKG

KPKG

**Fig. S5.** Abundance of oral cavity viruses in the analyzed viromes. Virome fragment recruitment of oral cavity viruses is shown in the different graphs for each virome. Y axis is represented in Kilobases recruited from each virome (Kb), normalized by viral length (Kb) and Virome size (Gb) (KPKG). Only reads with ≥70 % identity, ≥70 % query coverage and viruses with ≥40% coverage (subject coverage) are considered. Title of graph indicates virome used as in Supplementary Table S2.
