# Supplementary material for: Deciphering the Human Virome with Single-Virus Genomics and Metagenomics
Source: Viruses. 2018 Mar 6;10(3):113. doi: 10.3390/v10030113 (PMC5869506; doi:10.3390/v10030113)
Supplement: Supplementary file 1 [file viruses-10-00113-s001.zip › Supplementary Information/Fig S6.docx]

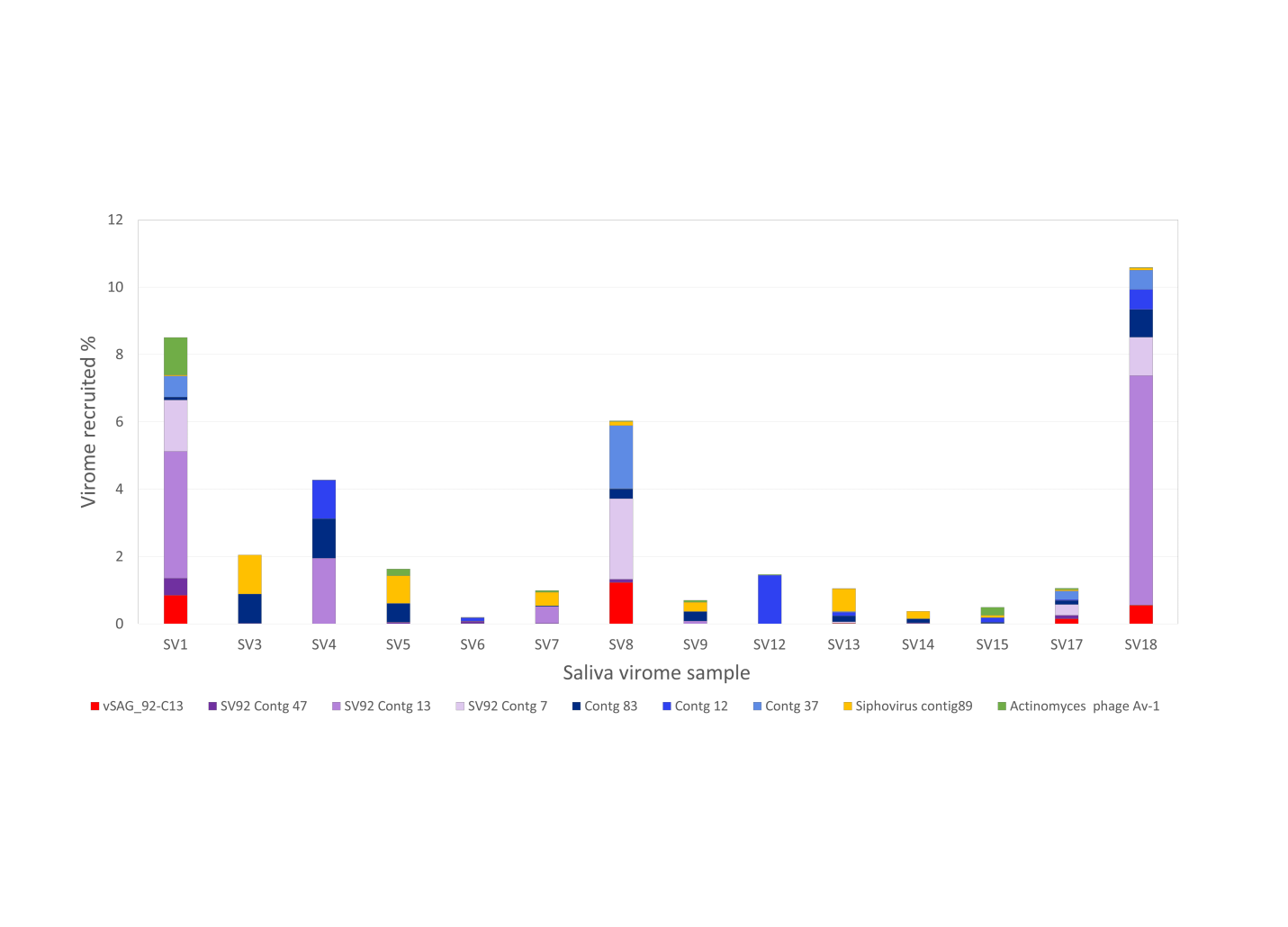


**Fig. S6**. Percentage of recruited virome for each one of those abundant viruses depicted in Figure 4. See methods for details of virome recruitment.
